# Effect of coconut oil on growth performance, carcass criteria, liver and kidney functions, antioxidants and immunity, and lipid profile of broilers

**DOI:** 10.1038/s41598-023-41018-3

**Published:** 2023-08-26

**Authors:** Mohamed S. Elewa, Diaa E. Abou-Kassem, Mohamed M. El-Hindawy, Mahmoud Madkour, Mohamed S. Elsharkawy, Mohamed Afifi, Mahmoud Alagawany

**Affiliations:** 1 Poultry Department, Faculty of Agriculture, Zagazig University, Zagazig, 44511 Egypt; 2 Animal Production Department, Faculty of Technology and Development, Zagazig University, Zagazig, 44511 Egypt; 3 Animal Production Department, National Research Centre, Dokki, 12622 Cairo Egypt; 4 Department of Biochemistry Faculty of Veterinary Medicine, Zagazig University, Zagazig, Egypt

**Keywords:** Immunology, Physiology

## Abstract

The aim of this study is to evaluate the beneficial effects of coconut essential oil on growth performance, carcass criteria, antioxidant status, and immune response of broiler chicks. A total of 192 un-sexed 7-days broiler chicks were divided into six treatment sets with four copies of 8 chicks per set. The groups were as follows: (1) basal diet (without additive), (2) basal diet plus 0.5 ml coconut essential oil/kg, (3) basal diet plus 1 ml coconut essential oil/kg, (4) basal diet plus 1.5 ml coconut essential oil/kg, (5) basal diet plus 2 ml coconut essential oil/kg and (6) basal diet plus 2.5 ml coconut essential oil/kg. The results showed that the most prevalent compound in coconut oil is 6-Octadecenoic acid (oleic acid) representing 46.44% followed 2(3H)-Furanone, dihydro-5-pentyl- (CAS) (11.36%), Hexadecanoic acid (CAS) (4.71%), and vanillin (2.53%). Dietary 1 and 1.5 ml of coconut oil improved significantly the body weight and gain of broiler chickens. Dietary supplementation of 1 ml of coconut oil improved significantly liver function compared to control and other treatment groups. The supplementation with 1 ml coconut oil significantly reduced TG and VLDL compared to control and other treatment groups, while no significant differences in TC, HDL, and LDL due to dietary coconut oil. The present findings showed that dietary coconut oil with 1 and 1.5 ml/kg feed improved significantly antioxidants status through increased antioxidant enzymes like SOD and GSH while decreasing significantly MDA levels compared to control and other treatment groups. Therefore, it was concluded that the diets of broiler chickens could be fortified with coconut oil with 1 or 1.5 ml to improve the growth, feed utilization, and antioxidant status of broiler chickens.

## Introduction

In recent years, there is great attention to the use of natural feed additives in poultry production as a global demand. The successful application of herbal growth promoters increases the profitability of the poultry industry by enhancing feed efficiencies and health conditions^1,2^. The secondary metabolites of herbal plants, including phenolic compounds, saponins, and essential oils, are linked to some of the health benefits^3,4^.

Coconut oil is saturated oil, and medium-chain fatty acid (MCFA) represents about 60% of its total fatty acid composition that contains a chain length of 6 to 12 carbon atoms, which are absorbed directly into the portal circulation without re-esterification in the intestinal cells^5^. The antibacterial properties, antioxidant activity, and anti-inflammatory effect of coconut oil make it a valuable feed additive in poultry feed^6,7^. The main natural antioxidants found in coconut oil include capric acid, tocotrienols, and lauric acid.

In broiler chickens, coconut oil significantly increased the growth through the 1–21-days period (9.9%) compared the fish oil-diet^8^. The supplementation with coconut oil significantly increased superoxide dismutase (SOD) activity compared to the fish oil group. Besides, coconut oil-supplemented rations significantly decreased plasma malondialdehyde (MDA) compared to the fish oil diet^8^. While, Wang et al.^9^ stated that a coconut oil -supplemented diet has no impact on body weight gain (BWG), feed intake (FI) and feed conversion ratio (FCR). In addition, coconut oil improves the digestion of fats and the performance index during coccidiosis infection^9^.

It is hypothesized that the dietary addition of coconut oil is expected to exert beneficial effects on broiler chicks. Thus, this study aimed to determine the impact of coconut essential oil on growth performance, carcass criteria, liver and kidney function, antioxidant and immunity, and lipid profile of broiler chicks.

## Material and methods

### Ethical statement

The experiment was accepted by the Ethics Committee of the Local Experimental Animals Care Committee and performed under the guidelines of the Department of Poultry, Faculty of Agriculture, Zagazig University, Egypt (ZU-IACUC/2/F/56/2021). The study was conducted following ARRIVE guidelines.

### Animals, design and diets

One hundred ninety-two broiler chicks were divided into six groups sets with four replicates of 8 chicks per set. The groups were as follows: (1) basal diet (control), (2) basal diet plus 0.5 ml coconut essential oil/kg, (3) basal diet plus 1 ml coconut essential oil/kg, (4) basal diet plus 1.5 ml coconut essential oil/kg, (5) basal diet plus 2 ml coconut essential oil/kg and (6) basal diet plus 2.5 ml coconut essential oil/kg. We provided standard broiler rations (National Research Council, 1994). Water and food were available freely. Two phases of commercial diets were administered: the beginning (0–3 weeks) and the finishing phase (4–5 weeks). Table 1 indicates the form and structure of trade broiler rations.

**Table 1:** Composition and chemical analysis of the basal diets (starter and finisher diets).

Items	Starter	Finisher
Ingredients (%)		
Yellow corn	50.53	59.25
Soybean meal	38.50	33.50
Soybean oil	0.30	1.40
Bran	7.50	3.00
Mono calcium P	1.00	0.90
Limestone	1.30	1.20
Vit-min premix*	0.30	0.30
NaCl	0.30	0.30
DL methionine	0.11	0.10
L-Lysine	0.11	0.01
Choline chloride 60%	0.05	0.04
Total	100	100
Calculated analysis** (%)		
CP	23.00	20.00
ME Kcal/kg diet	2900	3100
Ca	1.00	0.90
P (available)	0.48	0.45
Lysine	1.40	1.20
M + C	0.92	0.72
CF	3.43	2.88
Linoleic acid	1.50	20.40

*Growth vitamin and Mineral premix Each 2.5 kg consists of Vit A 12,000, 000 IU; Vit D3, 2000, 000 IU; Vit. E. 10 g; Vit k3 2 g; Vit B1, 1000 mg ; Vit B2, 49 g ; Vit B6, 105 g; Vit B12, 10 mg; Pantothenic acid, 10 g; Niacin, 20 g , Folic acid , 1000 mg ; Biotin, 50 g; Choline Chloride, 500 mg, Fe, 30 g; Mn, 40 g; Cu, 3 g; Co, 200 mg; Si, 100 mg and Zn , 45 g.

**Calculated according to NRC (1994).

### Gas chromatography/ mass spectrometry examination of coconut essential oil

According to Adams^10^, the components (major and minor) of coconut essential oil were identified and measured by gas chromatography (Agilent Technologies 6890 series, Wilmington, DE, USA).

### Growth parameters, blood sampling and carcass properties

To determine weight changes and body weight growth, chick weights at ages 0, 3, and 5 weeks were recorded. At ages 1, 3, and 5 weeks, feed consumption and conversion were also measured. At the end of the experiment, chickens (twenty four birds; 4 birds randomly possessed per group) were weighed and then were anesthetized by using intramuscular injection with 1 ml/kg of ketamine xylazine mixture (2:1) and slaughtered by sharp knife to complete bleeding, and their blood was taken in sterile tubes. The samples were then centrifuged after clotting at 3500 rpm (2328.24 G) for 15 min, and serum was kept at -20 °C until biochemical analysis. Weights of the heart, gizzard, and liver were measured and given as grams per kilogramme of killing weighing (KW). Weighing measurements were taken for the carcass, dressed, and giblet; (Carcass weight + Giblet weight)/Lives body weight was used to calculate the dressed weight.

### Biochemical parameters

The collected sera were used to determine total protein (TP), albumin (ALB), liver function represent as aspartate aminotransferase (AST), alanine aminotransferase (ALT), kidney function represent as creatinine, urea grades, lipid profile like total cholesterol (TC), high-density lipoprotein (HDL), cholesterol, triglyceride (TG). Also immune response such as immunoglobulin G (IgG) and immunoglobulin M (IgM) and antioxidants (superoxide dismutase; SOD, reduced glutathione; GSH and malondialdehyde; MDA) were measured using commercial diagnostic tools from Biodiagnostic Co. (Giza, Egypt). Low-density lipoprotein (LDL) cholesterol was studied via the Friedewald et al.^11^ model: $LDL = TC - HDL - TG/5$.

### Statistical analysis

One-way ANOVA was used to statistically examine the variances between sets. SPSS® (2008) statistical software v.11.0 was used for all analyses. Duncan's Multiple Range Test was used to determine the significant intergroup differences^12^.

## Results

### Bioactive components and essential oils in coconut oil

Table 2 displays the results of the analyses for coconut oil using gas chromatography and mass spectrometry, along with the retention times and peak area percentages. The most prevalent compound in coconut oil is 6-Octadecenoic acid (oleic acid) representing 46.44% followed 2(3H)-Furanone, dihydro-5-pentyl- (CAS) (11.36%), Hexadecanoic acid (CAS) (4.71%), and vanillin (2.53%).

**Table 2:** Bioactive chemical constituents assigned in coconut essential oil by GC–MS analysis.

No	Bioactive chemical constituents	Mass Weight (MW)	Retention Time (RT) (min)	Molecular Formula	Area%
1	2(3H)-Furanone, dihydro-5-pentyl- (CAS)	156	15.90	C9H16O2	11.36
2	Vanillin	152	16.81	C8H8O3	2.53
3	2H-1-Benzopyran-2-one (CAS)	146	17.67	C9H6O2	0.75
4	l-( +)-Ascorbic acid 2,6-dihexadecanoate	652	29.05	C38H68O8	4.71
5	Hexadecanoic acid (CAS)	256	29.05	C16H32O2	4.71
6	6-Octadecenoic acid	282	32.93	C18H34O2	46.44
7	17-Pentatriacontene	490	36.59	C35H70	2.26
8	Benzaldehyde, 4-hydroxy-3-methoxy- (CAS)	152	16.81	C8H8O3	2.53
9	4-PENTYLBUTAN-4-OLIDE	156	15.90	C9H16O2	11.36
10	FLAVONE 4′-OH,5-OH,7-DI-O-GLUCOSI DE	594	44.64	C27H30O15	0.93

### Growth parameters

Live body weight and weight gain data are presented in Table 3. The supplementation with 1 and 1.5 ml of coconut oil improved significantly body weight and gain (quadratic *p* < 0.01). In contrast, 2.5 ml of coconut oil impaired significantly body weight and gain. With the same trend, dietary supplementation of 1 ml of coconut oil improved significantly FCR (Table 4) while 2.5 ml level increased FCR compared to other levels and control groups. Feed intake increased significantly due to dietary supplementation of 2.5 ml of coconut oil (Table 4).

**Table 3:** Live body weight (LBW) and body weight gain (BWG) of broiler chicks as affected by dietary levels of coconut essential oil.

Treatments	LBW^1^ (g)			BWG^1^ (G)		
	1 week	3 weeks	5 weeks	1–3 weeks	3–5 weeks	1–5 weeks
Coconut oil level (ml/kg)						
0	170.40	809.05	1922.70	45.62	79.55	62.58
0.50	169.50	800.98	1861.17	45.11	75.73	60.42
1.00	168.67	852.32	2142.83	48.83	92.18	70.51
1.50	173.33	829.22	2145.00	46.85	93.98	70.42
2.00	173.17	829.22	1820.00	46.86	70.77	58.82
2.50	170.00	807.58	1653.17	45.54	60.40	52.97
SEM	0.59	8.40	46.01	0.58	3.17	1.63
*p* value						
Linear	0.256	0.665	0.059	0.710	0.053	0.055
Quadratic	0.594	0.202	0.001	0.202	0.002	0.001

Means in the same column significantly different (*p* < 0.05).

^1^*LBW* live body weight, *BWG* Body weight gain.

^2^*SEM* standard error means.

^3^*p* value (Linear and quadratic).

**Table 4:** Feed intake (FI) and feed conversion ratio (FCR) of broiler chicks as affected by dietary levels of coconut essential oil.

Treatments	FI^1^ (g)			FCR^1^ (g feed/ g gain)		
	1–3 weeks	3–5 weeks	1–5 weeks	1–3 weeks	3–5 weeks	1–5 weeks
Coconut oil level (ml/kg)						
0	66.51	95.16	80.83	1.46	1.20	1.29
0.50	69.11	104.55	86.83	1.55	1.43	1.44
1.00	70.42	108.15	89.29	1.44	1.18	1.27
1.50	72.60	114.29	93.44	1.56	1.22	1.33
2.00	70.81	98.75	84.78	1.51	1.41	1.45
2.50	72.08	114.02	93.05	1.58	1.94	1.78
SEM	1.08	2.12	1.38	0.33	0.07	0.04
*p* value						
Linear	0.104	0.005	0.004	0.373	0.002	0.001
Quadratic	0.450	0.129	0.128	0.891	0.013	0.009

Means in the same column significantly different (*p* < 0.05).

^1^*FI* feed intake, *FCR* feed conversion ratio.

^2^*SEM* Standard Error Means.

^3^*p* value (Linear and quadratic).

### Carcass measurements

No significant changes were seen among all the carcass parameters examined (dressing, liver, gizzard, and giblets) in response to coconut oil (Table 5).

**Table 5:** Carcass traits of broiler chickens as affected by dietary levels of coconut essential oil.

Treatments	Carcass traits (%)					
	Carcass%	Heart %	Liver%	Gizzard%	Giblets%	Dressing%
Coconut oil level (ml/kg)						
0	79.33	0.46	2.48	1.91	4.85	84.18
0.50	78.57	0.46	2.67	1.72	4.84	83.42
1.00	78.34	0.48	2.34	1.77	4.58	82.93
1.50	78.35	0.40	2.26	1.91	4.57	82.92
2.00	76.74	0.50	2.36	1.86	4.73	81.47
2.50	74.82	0.56	2.84	2.06	5.46	80.29
SEM	0.51	0.01	0.08	0.04	0.10	0.49
*p* value						
Linear	0.008	0.089	0.688	0.262	0.288	0.014
Quadratic	0.301	0.084	0.148	0.104	0.038	0.588

Means in the same column significantly different (*p* < 0.05).

^2^*SEM* Standard Error Means.

^3^*p* value (Linear and quadratic).

### Liver and kidney functions

Regarding liver function (Table 6), dietary supplementation of 1 ml of coconut oil improved significantly (linear *p* < 0.01) liver function in comparison to the control and other treatment groups. The addition of coconut oil to broiler diets has no significant effect on total protein, albumin, globulin, and creatinine. The supplementation with coconut oil linearly increased ALT compared to the control group (*p* = 0.002). Dietary coconut oil with 0.5, 1, 2 and 2.5 ml /kg significantly reduced AST compared to the control group (*p* = 0.007).

**Table 6:** Liver and kidney functions of broiler chicks as affected by dietary levels of coconut essential oil.

Treatments	Liver and kidney functions						
	TP (mg/dL)	ALB (mg/dL)	Glob (mg/dL)	ALT (U/L)	AST (U/L)	Creatinine (mg/dL)	Urea (mg/dL)
Coconut oil level (ml/kg)							
0	2.22	1.27	0.94	8.25	231.86	0.36	4.63
0.50	2.12	1.10	1.02	11.58	222.23	0.39	2.28
1.00	2.36	1.38	0.98	15.47	176.77	0.36	3.10
1.50	2.18	1.21	0.98	10.22	232.23	0.38	2.51
2.00	2.33	1.19	1.14	14.37	191.10	0.38	4.36
2.50	2.50	1.33	1.17	16.09	137.80	0.46	5.21
SEM^2^	0.08	0.04	0.05	0.87	10.42	0.01	0.31
*p* value^3^							
Linear	0.381	0.766	0.232	0.002	0.007	0.103	0.338
Quadratic	0.646	0.625	0.694	0.521	0.325	0.260	0.002

Means in the same column significantly different (*p* < 0.05).

^1^*TP* total protein, *ALB* albumin, *Glob* globulin, *ALT* alanine aminotransferase, *AST* aspartate aminotransferase.

^2^*SEM* Standard Error Means.

^3^*p* value (Linear and quadratic).

### Lipid profile

The supplementation with 1 ml coconut oil significantly reduced TG and VLDL compared to control and other treatment groups (Table 7) while no significant differences in TC, HDL, and LDL due to dietary coconut oil.

**Table 7:** Blood lipid profile of broiler chicks as affected by dietary levels of coconut essential oil.

Treatments	Lipid profile^1^				
	TC (mg/dL)	TG (mg/dL)	HDL (mg/dL)	LDL (mg/dL)	VLDL (mg/dL)
Coconut oil level (ml/kg)					
0	140.62	44.83	47.79	83.87	8.96
0.50	141.40	43.20	47.82	84.94	8.64
1.00	144.50	35.35	49.66	87.77	7.06
1.50	123.78	38.68	47.05	68.99	7.73
2.00	163.57	51.63	48.87	104.37	10.32
2.50	149.73	55.53	48.69	89.94	11.10
SEM^2^	4.37	2.20	0.42	4.17	0.44
*p* value^3^					
Linear	0.317	0.086	0.539	0.434	0.085
Quadratic	0.445	0.019	0.892	0.582	0.019

Means in the same column significantly different (*p* < 0.05).

^1^*TC* total cholesterol, *TG* triglycerides, *HDL* high density lipoprotein, *LDL* low density lipoprotein, *VLDL* very low density lipoprotein.

^2^*SEM* Standard Error Means.

^3^*p* value (Linear and quadratic).

### Antioxidant status and immunity

Regarding immune response and antioxidants status (Table 8), the present findings showed that dietary coconut oil with 1 and 1.5 ml/kg improved significantly antioxidants status through increased antioxidant enzymes like SOD and GSH while decreasing significantly MDA levels compared to control and other treatment groups. However, coconut supplementation has no significant effect on immune response.

**Table 8:** Antioxidants indices and immune parameters of affected by dietary levels of coconut essential oil.

Treatments	Antioxidants indices and immune parameters^1^				
	SOD (ng/mL)	MDA (ng/mL)	GSH (ng/mL)	IgG (ng/mL)	IgM (ng/mL)
Coconut oil level (ml/kg)					
0	49.40	6.45	104.00	263.00	181.00
0.50	113.67	1.72	62.00	302.67	164.00
1.00	150.33	1.64	51.39	254.67	143.67
1.50	136.00	1.26	83.00	180.33	90.33
2.00	135.00	0.57	150.00	58.33	20.24
2.50	151.67	0.54	174.33	289.33	172.67
SEM^2^	10.42	0.62	12.68	42.10	27.60
*P* value^3^					
Linear	< 0.001	< 0.001	0.012	0.464	0.330
Quadratic	0.007	0.018	0.007	0.578	0.358

Means in the same column significantly different (*p* < 0.05).

^1^*SOD* superoxide dismutase, *MDA* malondialdehyde, *GSH* Glutathione reduced, *IgG* immunoglobulin G, *IgM* immunoglobulin M.

^2^*SEM* Standard Error Means.

^3^*p* value (Linear and quadratic).

## Discussion

Our findings showed that dietary coconut oils up to 1.5 ml/kg significantly improved body weight and weight gain compared to a control group, this might be due to the bioactive compounds in coconut oil^13,14^. On the same context, use of coconut oil in broiler diets increased the growth rate during the 1–21-days period (9.9%) compared the fish oil-diet^8^. Moreover, it is suggested that that plant-derived essential oils can increase appetite. The digestion and absorption of nutrients in the gut are also improved by herbal supplements^15^. Several studies demonstrated the promotional effects of dietary coconut oil on growth performance in broilers^13^, and Japanese quail^16^. Moreover, dietary 2% coconut oil improved growth performance in broiler chicks challenged with coccidiosis^17^. However, dietary coconut has no effect on weight gain of broiler chickens^18^. In the same line dietary coconut at 1% had not negative effect in European quail performance^19^. These discrepancies in the results may be due the dietary level or the extraction process. The present findings showed that the best FCR was obtained by 1 ml of coconut , while the worst level was 2.5 ml level, the reason for that might due to increase the level of caprylic acid supplementation that affect feed conversion in broiler chickens^20^, however the mode of action of caprylic acid in affecting FCR is not clear .

Inclusion of 1 ml coconut oil in to broiler diets improved liver function. It could be due to the medium-chain fatty acids (MCFA), particularly lauric acid, in coconut oil's components, which have antioxidant and anti-inflammatory characteristics^21^. These findings point to a protective effect of PUFA on the integrity of hepatic cell membranes, which may be caused by increased phospholipids, which are a crucial component of cell membrane integrity and contain two hydrophobic long-chain fatty acids LCFA^22,23^.

Regarding to lipid profile, our findings showed that coconut oil inclusion at 1 ml coconut oil reduced significantly TG and VLDL. Our results agree with Attia et al.^8^ who found that dietary coconut oil has a positive effect on lipid profile in broiler chickens. Our finding showed that the lack of an impact on HDL cholesterol, demonstrated that MCFA in the form of coconut oil didn't lower cholesterol by bringing LDL cholesterol to the liver where it could be processed again, but rather by some other mechanisms^18^. Due to their effect on raising HDL-C while lowering LDL-C, the hazardous lipoprotein segment, UFA and beneficial PUFA have been shown in previous research to have desirable and healthful effects on plasma lipids^24–26^.

The present findings showed that dietary coconut oil with 1 and 1.5 ml /kg improved significantly antioxidants status through increased antioxidant enzymes like SOD and GSH while decreasing significantly MDA levels in comparison to the control and other groups. These results agree with Attia et al.^8^ who found that dietary supplementation of coconut oil decreased plasma MDA compared to the fish oil diet in broiler chickens. The main natural antioxidants found in coconut oil include capric acid, tocotrienols, and lauric acid. It has been reported that coconut oil as a source of lauric acid has an antioxidant activity in broilers diet^27^. Moreover, dietary coconut oil in rabbit diet improved GSH and SOD^12^, improved the antioxidant status in coconut oil supplemented diets might due to the bioactive ingredients that existing in coconut like higher saturated fatty acids and polyphenolic compounds, that have antioxidant properties^28^. Boosting the secretion of digestive enzymes, endocrine function, immune function, and antioxidant status are just a few of the multiple ways that phytogenic supplements may perform^29,30^. Our results indicated that broilers' growth performance and health condition could be improved in the future by using coconut oil as a natural antioxidant.

In the present study, dietary supplementation with coconut oil has not significant effect on immune response; these results disagree with previous one in broilers^24^ they found that the coconut-enriched diets in broiler chickens significantly increased α1-globulin. Moreover, El-Abasy et al.^21^ suggested that including dietary coconut (2%) in rabbit diets may have a positive impact on the animals' health and immunological functions. With the same trend, El Kholy et al.^31^ found that dietary supplementation of 1.5 or 2% of coconut oil in Domyati ducklings diets increased plasma immunoglobulin levels, which collectively suggested that the immunological response had improved. The non- significant effect of coconut oil as immune stimulator in our study may be due to unstressed condition that birds reared in.

## Conclusion

Our findings showed that dietary coconut oil with 1 and 1.5 ml improved significantly body weight, gain, and FCR in broiler chickens. Also, the antioxidant status of broiler chickens was improved due to dietary coconut oil. However, the immune response has not affected by coconut oil supplementation. The supplementation with 1 ml coconut oil reduced TG and VLDL compared to control and other treatment groups. Therefore, the diets of broiler chickens could be fortified with coconut oil with 1 or 1.5 ml to improve growth, feed utilization, and antioxidant status of broiler chickens.

## Data Availability

The datasets used and/or analyzed during the current study are available from the corresponding author upon reasonable request.

## Abbreviations

TG: Triglyceride
HDL: High density lipoprotein
LDL: Low density lipoprotein
VLDL: Very low density lipoprotein
SOD: Superoxide dismutase
GSH: Reduced glutathione
MDA: Malondialdehyde
BWG: Body weight gain
FI: Feed intake
FCR: Feed conversion ratio
MCFA: Medium-chain fatty acid
